# A robust COVID-19 mortality prediction calculator based on Lymphocyte count, Urea, C-Reactive Protein, Age and Sex (LUCAS) with chest X-rays

**DOI:** 10.1038/s41598-022-21803-2

**Published:** 2022-10-29

**Authors:** Surajit Ray, Abhirup Banerjee, Andrew Swift, Joseph W. Fanstone, Michail Mamalakis, Bart Vorselaars, Craig Wilkie, Joby Cole, Louise S. Mackenzie, Simonne Weeks

**Affiliations:** 1 School of Mathematics and Statistics, University of Glasgow, Glasgow, G12 8QQ UK; 2 Institute of Biomedical Engineering, Department of Engineering Science, University of Oxford, Oxford, OX3 7DQ UK; 3 Department of Infection, Immunity and Cardiovascular Disease, University of Sheffield, Sheffield, S10 2RX UK; 4 Brighton and Sussex Medical School, Brighton, BN1 9PX UK; 5 School of Computer Science, University of Sheffield, 211 Portobello, Sheffield City Centre, Sheffield, S1 4DP UK; 6 School of Mathematics and Physics, University of Lincoln, Brayford Pool, Lincoln, LN6 7TS UK; 7 School of Applied Sciences, University of Brighton, Brighton, BN2 4AT UK

**Keywords:** Computational models, Computational biology and bioinformatics, Biomarkers, Diseases, Health care, Medical research

## Abstract

There have been numerous risk tools developed to enable triaging of SARS-CoV-2 positive patients with diverse levels of complexity. Here we presented a simplified risk-tool based on minimal parameters and chest X-ray (CXR) image data that predicts the survival of adult SARS-CoV-2 positive patients at hospital admission. We analysed the NCCID database of patient blood variables and CXR images from 19 hospitals across the UK using multivariable logistic regression. The initial dataset was non-randomly split between development and internal validation dataset with 1434 and 310 SARS-CoV-2 positive patients, respectively. External validation of the final model was conducted on 741 Accident and Emergency (A&E) admissions with suspected SARS-CoV-2 infection from a separate NHS Trust. The LUCAS mortality score included five strongest predictors (Lymphocyte count, Urea, C-reactive protein, Age, Sex), which are available at any point of care with rapid turnaround of results. Our simple multivariable logistic model showed high discrimination for fatal outcome with the area under the receiving operating characteristics curve (AUC-ROC) in development cohort 0.765 (95% confidence interval (CI): 0.738–0.790), in internal validation cohort 0.744 (CI: 0.673–0.808), and in external validation cohort 0.752 (CI: 0.713–0.787). The discriminatory power of LUCAS increased slightly when including the CXR image data. LUCAS can be used to obtain valid predictions of mortality in patients within 60 days of SARS-CoV-2 RT-PCR results into low, moderate, high, or very high risk of fatality.

## Introduction

The UK National Health Service (NHS) experienced unprecedented pressure due to the recurrent surges of coronavirus disease 2019 (COVID-19) cases caused by the SARS-CoV-2 virus. To date, more than two million confirmed cases in the UK have forced healthcare professionals to face complex decisions on how to effectively triage patients on admission who may need acute care. Admissions to hospital have increased from 6894 to 8431 in the seven days ending 8th March 2022 in the UK where routine lateral flow testing by the general public is no longer required and are ‘incidentally diagnosed’^1^. Conditions remain strained, and there is a need to develop risk tools to enable accurate and rapid triaging of SARS-CoV-2 positive patients. While prognostic tools are available, there is an ongoing need to develop tools that enable healthcare professionals to prevent unnecessary hospital admission and also work in combination with other predictive models commonly used in practice. Any new model must support the healthcare professional’s experiential knowledge and clinical reasoning^2^ in both patient care management and allocation of limited healthcare resources.

In hospitals, the main method used to detect COVID-19 is the reverse transcription-polymerase chain reaction (RT-PCR), and the severity of the infection determined by blood markers and chest radiological imaging such as X-ray and computed tomography (CT)^3–5^ scans. While CT is a sensitive tool, it is not used for periodic monitoring of patients. The use of x-ray is widespread and is used routinely to determine severity of lung injury. Its use has been included in numerous prognostic tools^6^, and in combination with other blood markers such as D-dimer, white blood count, and neutrophils^7^. However, some biomarkers such as D-dimer are not being routinely measured during hospital admission and triage.

Predictive models such as the National Early Warning Score 2 (NEWS2) is widely used in practice to observe and identify deteriorating patients^8,9^. Although favourable amongst healthcare professionals^10^, this predictive tool has caused increased false trigger rates for SARS-CoV-2 positive patients^11,12^. Another familiar tool in practice in the UK is the Acute Physiology and Chronic Health Evaluation II (APACHE II), which is a validated intensive care unit (ICU) scoring tool used to estimate ICU mortality. However, it has underestimated the mortality risk in SARS-CoV-2 positive patients^13^. Several published models have aimed to meet the clinician’s need to stratify high-risk SARS-CoV-2 positive patients; but they are yet to be widely clinically implemented or had poor clinician feedback^14^.

There are varying reports of changes in the full blood count, with it being reported that 80% of COVID-19 patients with severe symptoms and 20% of mild cases presented with lymphopenia^15^, which indicates that a low lymphocyte count is not an accurate marker taken alone^16,17^. Many studies have suggested the use of Interleukin 6 (IL6)^16^, Interleukin 10 (IL10)^16^, ferritin^16^, and D-Dimer^7,17^, since they are good discriminators of disease prognosis; but they are not routinely measured upon admission to hospital.

Here, we aim to avoid ‘clinical resistance’ by developing and validating a new simple and explainable prognostic tool that extends the widely used NEWS2 model^18^. This model combines clinical data, routine laboratory tests and chest x-ray (CXR) data to improve risk stratification and clinical intervention for SARS-CoV-2 infection. Studies have shown CXR results add value in the triage of patients who are SARS-CoV-2 positive^19^; however, the incremental prognostic value of CXR^6^ in addition to clinical data and routine laboratory tests remains to be consolidated in practice.

We used well-defined predictors and outcomes to limit model overfitting^20^. The model’s development and reporting adhered to the TRIPOD (transparent reporting of a multivariable prediction model for individual prediction or diagnosis) guidelines^21,22^ to publish a simple, user-friendly scoring system for adult patients admitted to hospital with SARS-CoV-2 infection. This resulted in improved integration of the best evidence into clinical care pathways that will assist clinicians in risk, patient, and resource management. The aim of this study was to develop a simple objective tool for risk stratification in SARS-CoV-2 infection by integrating results from rapid and routine clinical, laboratory and CXR image data that would easily support established triage practice in the hospital setting.

## Methods

### Sources of data

A prospective cohort study was conducted with the National COVID-19 Chest Imaging Database (NCCID)^23^ that collated clinical data from secondary and tertiary NHS Trusts across the UK with the aim to support SARS-CoV-2 care pathways (Supplementary Table 1). NHS staff submitted data for patients suspected of SARS-CoV-2 with a RT-PCR test that was positive or negative. The centralised data warehouse stored de-identified clinical data for those admitted to hospital between 23rd January 2020 and 7th December 2020, which was used for both the development and validation of the mortality risk tool.

To evaluate the tool’s generalised performance, a second dataset was used for external validation. The data extracted from the Laboratory Information Management System (LIMS) came from a large NHS Foundation Trust hospital in north-east England UK, which had not participated in the NCCID initiative. Patients who were admitted with suspected SARS-CoV-2 infection at the Accident and Emergency (A&E) Department between 1st March 2020 and 21st August 2020 were included. This population was representative of the adult general population and therefore patients aged 16 years and younger were excluded, as the laboratory data thresholds vary compared to adults. The datasets are retrospective and therefore consent is not possible.

### Participants

The three datasets (‘development’, ‘internal’ and ‘external validation’) consisted of data for adult patients with positive RT-PCR results for the SARS-CoV-2 virus. To mitigate the issues with the lower RT-PCR sensitivity and accuracy during the initial stages of the pandemic, NCCID provided an overall SARS-CoV-2 status that was derived from cumulative RT-PCR tests. This approach was also applied to identify SARS-CoV-2 positive patients in the external validation dataset.

**Figure 1:**
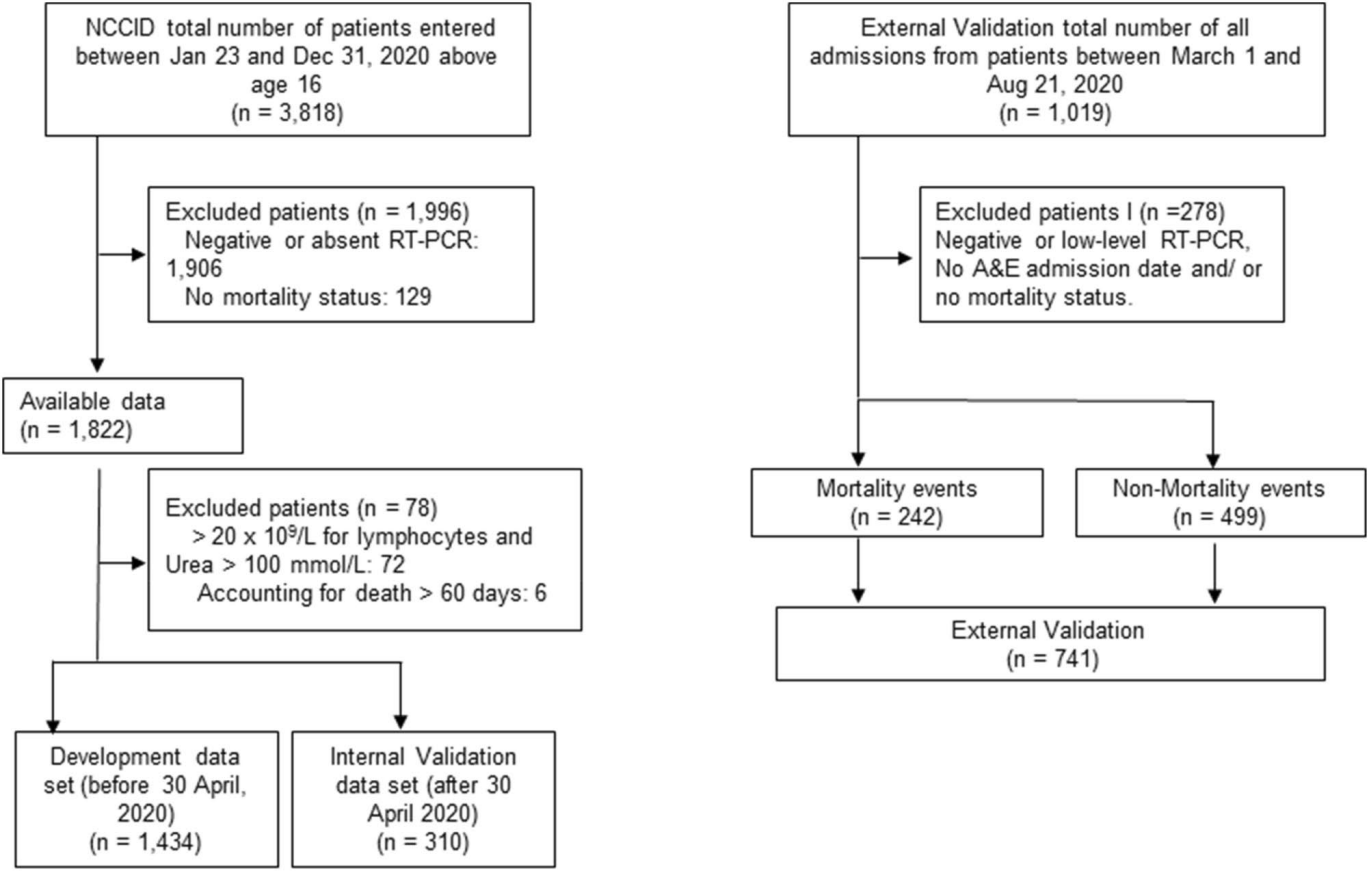
Patient flowchart with inclusion and exclusion criteria that established the development dataset as well as the internal and external validation datasets; n, number of patients in dataset.

To evaluate the tool’s predictive performance, the NCCID dataset was non-randomly split with the admissions before 30th April 2020 making up the development cohort (n = 1434) and admissions on or after 1st May 2020 making up the internal validation cohort (n = 310) (Fig. 1). The external validation cohort (n = 741) was created by identifying fatal outcomes within 60 days of A&E admission of all patients who were confirmed positive for SARS-CoV-2 infection by RT-PCR. This approach was rationalised since patients were more comparable on latent variables such as co-morbid illnesses that were not explicitly captured during the LIMS data extraction of laboratory results. In addition, the filtering on admitted patients increased the density of laboratory results across all the candidate predictors, which made the model less reliant on imputation.

### Outcome

The primary outcome of interest was in-hospital mortality within 60-days of a positive SARS-CoV-2 RT-PCR test. To identify this outcome, the date of death was followed up and entered by hospital staff as part of the NCCID initiative.

### Predictors

The NCCID dataset collected 38 clinical data points for each patient that served as candidate predictors. They were categorised into demographics, risk factors, past medical history (PMH), medications, clinical observations, chest imaging data, and laboratory parameters measured on admission. Chest X-ray (CXR) reports from the NCCID model development dataset and the external dataset were dichotomised as normal versus abnormal. All laboratory tests were measured before SARS-CoV-2 RT-PCR test and mortality date, which ensured these potential predictors were blinded to the outcome. The SARS-CoV-2 swab and RT-PCR results established the final COVID-19 status. In the event of deterioration, data points such as the NEWS2 score, Acute Physiology And Chronic Health Evaluation (APACHE) score, Intensive Therapy Unit (ITU) admission, intubation and mortality date were also recorded. Since the focus of the study was to identify predictors that were available at the point of admission, the ITU admission and intubation data were excluded in the final analysis.

From this extensive list, only the demographics, clinical observations, and laboratory parameters recorded at the time of admission for SARS-CoV-2 positive patients were included in the analysis as key predictors. Previous medical history, smoking status and current pharmaceutical interventions were not included in the analysis. This rationale was based on consultations with biomedical scientists and clinicians working in A&E departments to determine which rapid and routine laboratory tests would be clinically relevant candidate predictors, as well as previously published literature on prognostic factors associated with SARS-CoV-2 infection for the model development^24^.

To account for the risk of spurious selection or exclusion of important predictors in the model development, a minimum sample size of 380 was determined based on 10 outcome events per variable. Based on previous studies, a minimum sample size of 100 mortality events and 100 non-mortality events were required for external validation^25^.

### Statistical analysis

**Figure 2:**
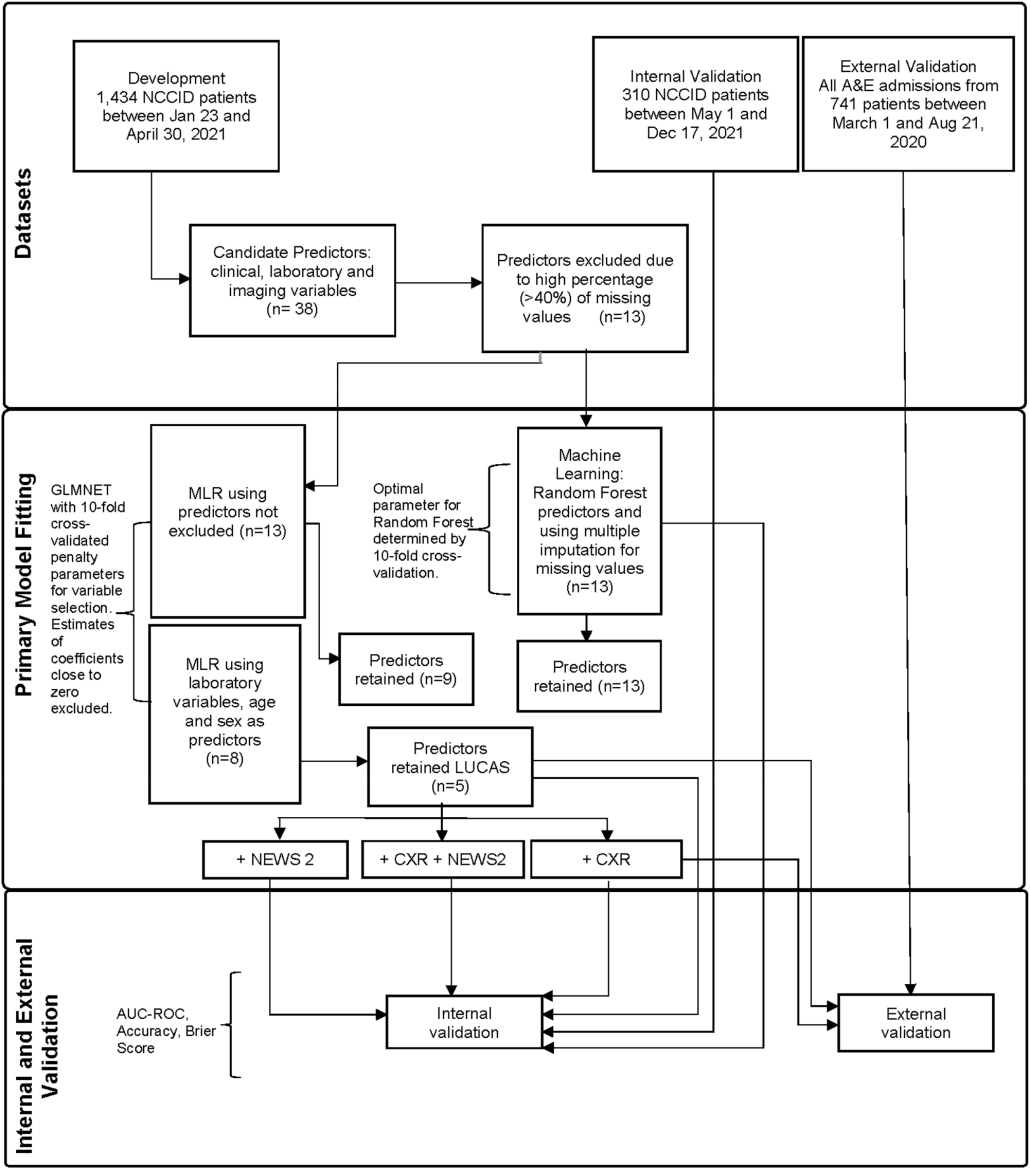
Model development, internal and external validation workflow. AUC-ROC, area under the receiver operating characteristic curve; GLMNET, Lasso and Elastic-Net Regularised Generalised Linear Models; MLR, multivariable logistic regression; LUCAS, model using Lymphocyte, Urea, CRP, Age, Sex; CXR, Chest X-ray; NEWS2, National Early Warning Score; *n*, the number of predictors.

Reliability of data was ensured by excluding patients from the development and internal validation datasets if information was missing on key characteristics, such as RT-PCR SARS-CoV-2 positive results at admission, and mortality dates outside the 60-day prediction interval (Fig. 2). Predictors with more than 40% missing values were also excluded from the modelling process (Supplementary Fig. 1). Missing data from the remaining predictors were handled using Multiple Imputation by Chained Equations (MICE), and ten different imputed datasets were combined using the Rubin rule^26^. Continuous predictors such as the laboratory tests and age were retained as continuous variables and not converted into categorical predictors using thresholds.

An initial multivariable logistic regression (MLR) model started with 13 predictors with less than 40% missing values (Lymphocyte, Urea, CRP, Age, Sex, White Cell Count (WCC), Creatinine, Platelets, Diastolic BP (Blood Pressure), Systolic BP, Temperature, Heart rate, PaO_2_). Elastic Net (GLMNET)^27^, weighted combination of Least Absolute Shrinkage and Selection Operator (LASSO), and ridge regression were used to select predictors of importance. The optimal value of the shrinkage estimator for LASSO and the relative weights for LASSO and ridge regression were chosen using a ten-fold cross validation using the R-package caret^28^. Additionally, the performance of the MLR model was compared with the performance of the Random Forest Machine Learning approach^29^. The variable selection and construction of the Random Forest was performed using ten-fold cross validation. In each case, the imbalance in the positive and negative training samples was accommodated using the Synthetic Minority Oversampling Technique (SMOTE).

A second separate MLR model took a more pragmatic approach in predictor selection. From the 13 predictors of the first MLR model, we used only the blood panel measurements (Lymphocyte, Urea, CRP, WCC, Creatinine, Platelets) along with age and sex. In addition, it was assessed whether the inclusion of either NEWS2 or CXR or both improved this model’s performance.

Each model’s performance was assessed using Area Under the Receiving Operating Characteristics Curve (AUC-ROC) with a 95% confidence interval. The calibration performance of each model was measured as the slope of the calibration curve. The internal and external validation of each model were assessed with resampling. The predictor selection and entire modelling process were repeated in 1,000 bootstrap samples to estimate the realistic predictive measures for future patient cohorts. An AUC-ROC of 0.5 indicated a prediction based on random chance, 0.7–0.8 was considered acceptable, above 0.8 demonstrated excellent performance, and a value of 1.0 showed a perfect prediction^30^. We also evaluated the goodness of fit using the Brier score^31^, which is a measure to quantify the closeness of the probabilistic predictions to the binary ground-truth class labels. The score varies between 0 and 1, with the lower score indicating superior performance. Finally, the accuracy of the models was evaluated using the standard cut-off value of 0.5.

The internal validation included the NEWS2 score, which was not available for the external validation dataset. The statistical package R (3.5.3) was used to perform all statistical analyses and implement the methods employed in the model development, validation workflow, and primary model fitting, followed by both internal and external validation.

### Data protection/Ethics

De-identified and pseudo-anonymised patient data were obtained from datasets, and the methods used were approved by the ethics committee as part of the existing Cardiac Magnetic Resonance Imaging (MRI) Database NHS Research Ethics Committee (REC) Integrated Research Application System (IRAS) Ref: 222349 and University of Brighton REC (8011). The need for informed consent was waived by the ethics committee due to retrospective nature of the study.

## Results

Overall, the NCCID database comprised of 3,818 patients (from 23rd January 2020 to 31st December 2020). The number of patients that met the inclusion and missingness criteria were 1434 and 310, for the development and internal validation datasets, respectively (Fig. 1). All available data on positive patients were used in the external validation dataset, consisting of 242 fatal (mortality) and 499 non-fatal (no mortality) cases. The development dataset initially included 38 predictors (continuous and categorical), which was followed by the removal of variables with ≥ 40% missingness. Summary statistics of median with interquartile range (IQR) for continuous variables and proportions for categorical variables were presented and compared to those for the internal validation and external validation datasets (Table 1).

**Table 1:** Comparison of development, internal and external dataset characteristics.

Characteristics	Development (n = 1434)	Internal validation (n = 310)	External validation (n = 741)
Data collection dates	23/01/2020–30/04/2020	01/05/2020–07/12/2020	01/03/2020–21/08/2020
Study design	Prospective cohort		Retrospective cohort
Setting	Secondary care hospital admissions from 19 sites across the UK		Secondary care hospital A&E admissions from one UK site
Inclusion criteria	All adult patients with a positive SARS-CoV-2 status (derived from cumulative RT-PCR test results) and data at the point of hospital admission		
Outcome	In-hospital mortality within 60 days of positive RT-PCR test		
Mortality *n* (%)	466 (33)	58 (18)	242 (33)
**Demographics**			
Ethnicity: White British, Irish or any other background *n* (%)	498 (35)	90 (29)	599 (81)
Ethnicity: Mixed white and Caribbean, Asian or any other background *n* (%)	6 (0.4)	0 (0)	8 (1)
Ethnicity: Asian or Asian British background *n* (%)	84 (6)	10 (3)	35 (5)
Ethnicity: Black or Black British background *n* (%)	78 (5)	2 (1)	45 (6)
Ethnicity: any other ethnicity *n* (%)	99 (7)	4 (1)	14 (2)
Ethnicity not stated or reported *n* (%)	669 (47)	204 (66)	40 (5)
Median age (IQR), [years]	73 (50)	74 (44)	76 (46)
Men *n* (%)	902 (63)	175 (57)	422 (57)
**Median clinical data on admission**			
Duration of symptoms (IQR) [days]	5 (7)	3 (6)	–
Respiratory rate (IQR) [breaths/min]	21 (7)	20 (6)	–
Heart rate (IQR) [beats/min]	90 (25.0)	89 (26.2)	–
PaO_2_ (IQR) [% room air]	93 (84.3)^a^	95 (6)	–
FiO_2_ (IQR) [oxygen flow rate if on O_2_]	21 (26)	4 (20.79)	–
NEWS2 score (IQR)	4 (4)^a^	3 (5.0)^a^	–
Diastolic BP (IQR) [mmHg]	74 (18.0)	74 (17.5)	–
Systolic BP (IQR) [mmHg]	130 (32.0)	131 (35.0)	–
Temperature (IQR) [^o^C]	40 (15.0)	38 (14.0)	–
**Median laboratory data on admission**			
WCC (IQR) [× 10^–9^/L]	7.2 (5.1)	8.0 (5.4)	8.1 (5.8)
Lymphocytes (IQR) [× 10^–9^/L]	0.9 (0.64)	1(0.74)	0.9 (0.8)
Platelets (IQR) [× 10^–9^/L]	212 (125.0)	229 (134.2)	220 (121.0)
D-Dimer (IQR) [µg/L]	–	–	1118 (1585)^a^
Fibrinogen (IQR) [g/L]	–	–	5.6 (2.1)
CRP (IQR) (mg/L)	85 (121.5)	46 (98.2)	62 (101.0)
Ferritin (IQR)^b^	541 (230.5)^a^	601 (188)^a^	483 (697)^a^
Urea (IQR) [mmol/L]	6.8 (6.0)	6.5 (5.3)	7.2 (5.5)
Creatinine (IQR) [µmol/L]	86 (57.0)	85 (49.0)	88 (49.5)
Troponin I (IQR) [ng/L]	12 (42.5)	15 (172)	–
Troponin T (IQR) [ng/L]	22 (40.5)^a^	17 (27)^a^	–
Chest X-ray data			
CXR (abnormal) *n *(%)	1006 (70)	172 (55)	361 (49)
**Past medical history**			
Hypertension *n* (%)	603 (42)	144 (46)	–
CVS disease *n* (%)	303 (21)	66 (21)	–
Diabetes mellitus type II n (%)	390 (27)	92 (30)	–
Lung disease *n* (%)	333 (23)	79 (26)	–
Chronic kidney disease *n* (%)	279 (20)	54 (17)	–
Current ACEi use *n* (%)	193 (14)	53 (17)	–
Current Angiotensin receptor blocker use *n* (%)	116 (8)	24 (8)	–
Current NSAID use *n* (%)	130 (9)	35 (11)	––
**Risk factors**			
Smokers *n* (%)	303 (21)	82 (27)	–
Median pack year history (IQR)	0 (26)	15 (40)	–
**Deterioration**			
ITU admission n (%)	181 (13)	38 (12)	–
Median APACHE score (IQR)	16 (9.25)	12.5 (9.75)	–
Intubation n (%)	143 (10)^a^	18 (6)^a^	–

RT-PCR, reverse transcription polymerase chain reaction; A&E, Accident and Emergency; WCC, White Cell Count; CRP, C-reactive protein.

^a^Indicates missing data values over 40% (detailed description of the proportion of missing values is presented in Supplementary Fig. 1).

^b^Indicates difference in units: internal dataset ng/ml and external dataset µg/L.

### Model development

**Figure 3:**
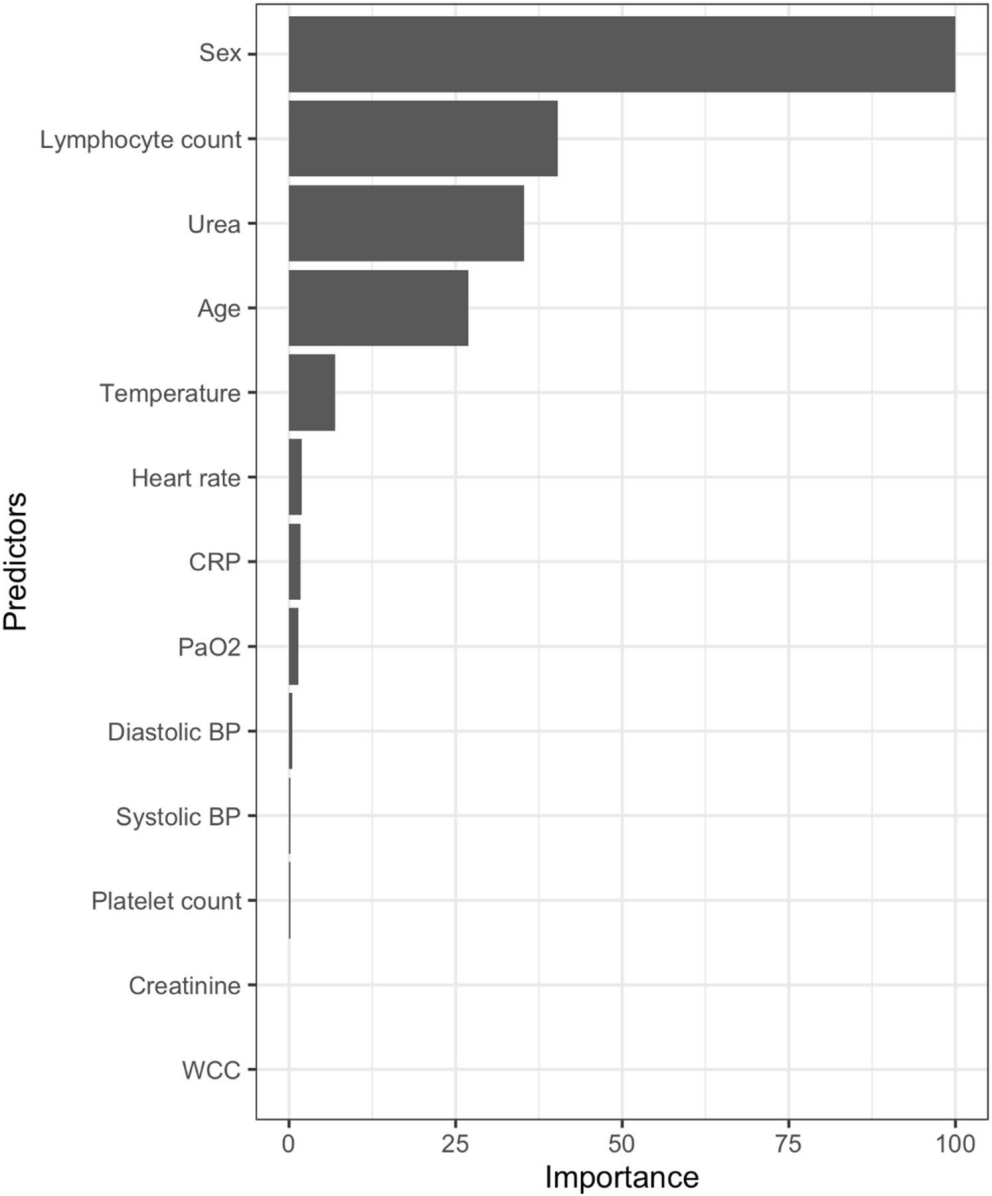
GLMNET (Lasso/Ridge) Variable importance plot with predictors having less than 40% missing values in the database. All patients are confirmed positive for SARS-CoV-2 by RT-PCR. The plot shows the importance of predictors in building the development predictive model. BP, blood pressure; PaO_2_, partial pressure of O_2_; WCC, white cell count; CRP, C-reactive protein.

From the 38 predictors in the NCCID database, we selected 13 predictor variables that were judged to be clinically important and had no more than 40% missing values. These predictors relate to demographics, clinical observations and laboratory parameters recorded at the time of admission (Fig. 2). The variable importance plot indicates the relative importance of these predictors (Fig. 3); the most important predictor was sex, then lymphocyte count, urea, followed by age. The GLMNET model selection criteria optimally chose 8 predictors based on ten-fold cross validation that resulted in the MLR model having an AUC-ROC of 0.759 (Table 2). In contrast, the Random Forest model used all 13 predictors, which increased the complexity of the prediction and achieved a slightly higher AUC-ROC value of 0.806. Thus, the simplicity and ease of fitting our MLR model make it a robust score which can be easily adaptable to new datasets.

**Table 2:** Best fit model by Lasso based sequential variable selection.

Model (no of predictors in initial model)	Predictors													Model performance					
	Lymphocyte	Urea	CRP	Age	Sex	WCC	Creatinine	Platelets	Diastolic BP	Systolic BP	Temperature	Heart Rate	PaO_2_	AUC-ROC	Sensitivity	Specificity	Precision	F1	Calibration
MLR (k = 8)	Y	Y	Y	Y	Y	N	N	N	N	N	Y	Y	Y	0.759	0.886	0.378	0.738	0.805	1.025
Random Forest (k = 13)	Y	Y	Y	Y	Y	Y	Y	Y	Y	Y	Y	Y	Y	0.806	0.890	0.661	0.838	0.863	1.021
LUCAS (k = 5)	Y	Y	Y	Y	Y	N	N	N	–	–	–	–	–	0.765	0.931	0.330	0.757	0.835	1.003

CRP, C-reactive protein; WCC, white cell count; AUC-ROC, area under the receiver operating characteristic curve; BP, blood pressure; PaO_2_, partial pressure O_2_; 'Y', retained in the model; ‘N’, excluded after statistical model selection; ‘–’, excluded from the initial model; k, number of predictors.

**Figure 4:**
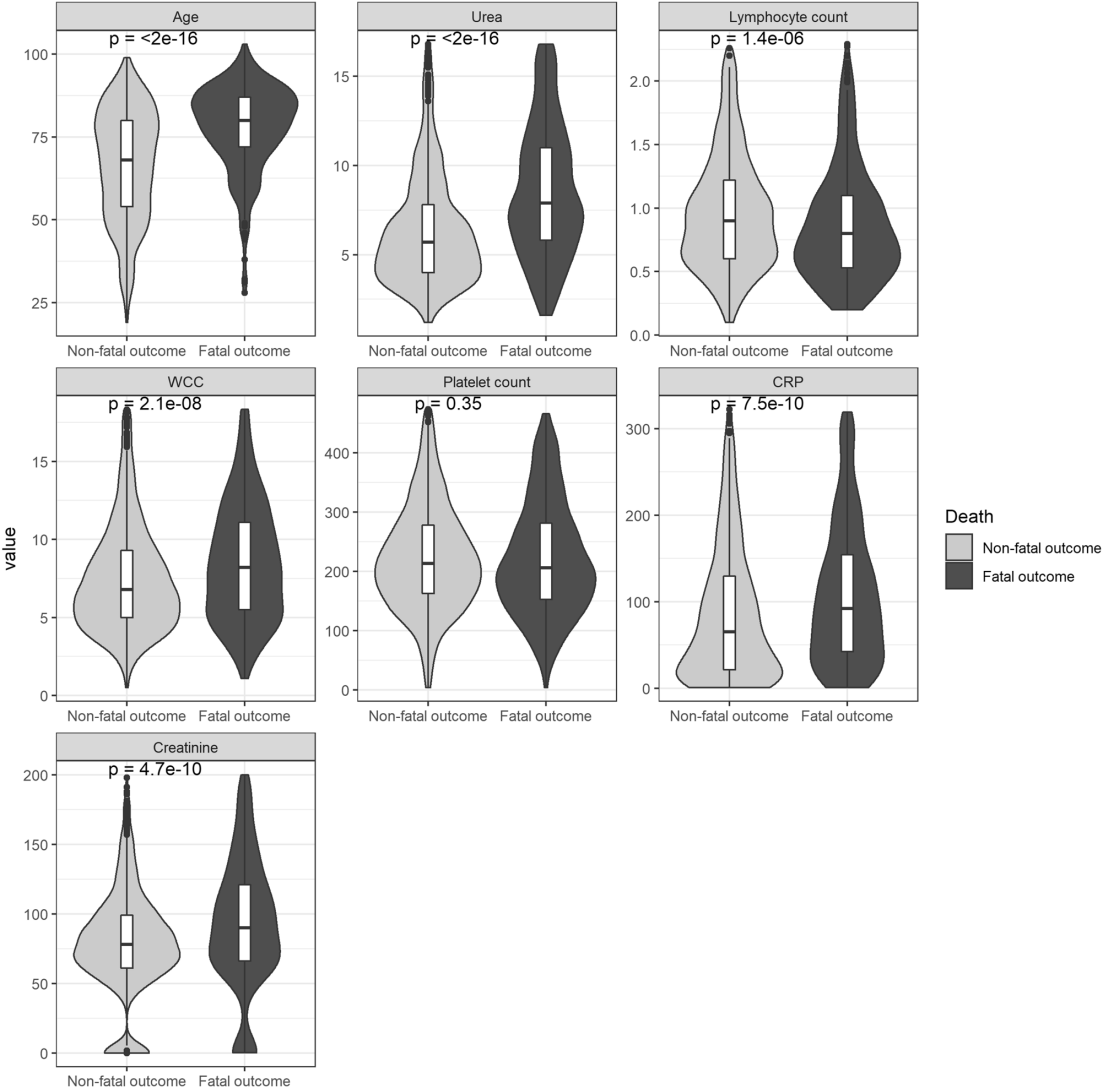
Violin plots of individual blood variables parameters and age, categorised by patient outcome. The boxplots showing median and 1st and 3rd quartiles of the predictor variables are overlayed on the violin plots. All NCCID patients tested positive by the RT-PCR test for SARS-CoV-2. WCC, white blood count; CRP, C-reactive protein. The *p*-values are tests of equality of population using the Wilcoxon rank-sum test, where *p* < 0.05 implies statistically significant difference between the populations.

The rapid blood test measurements, sex and age were recorded at the time of admission, and they parameterised our model. The relative difference of the laboratory parameters along with age, between patients with fatal and non-fatal outcomes, is presented as violin plots in Fig. 4.

**Figure 5:**
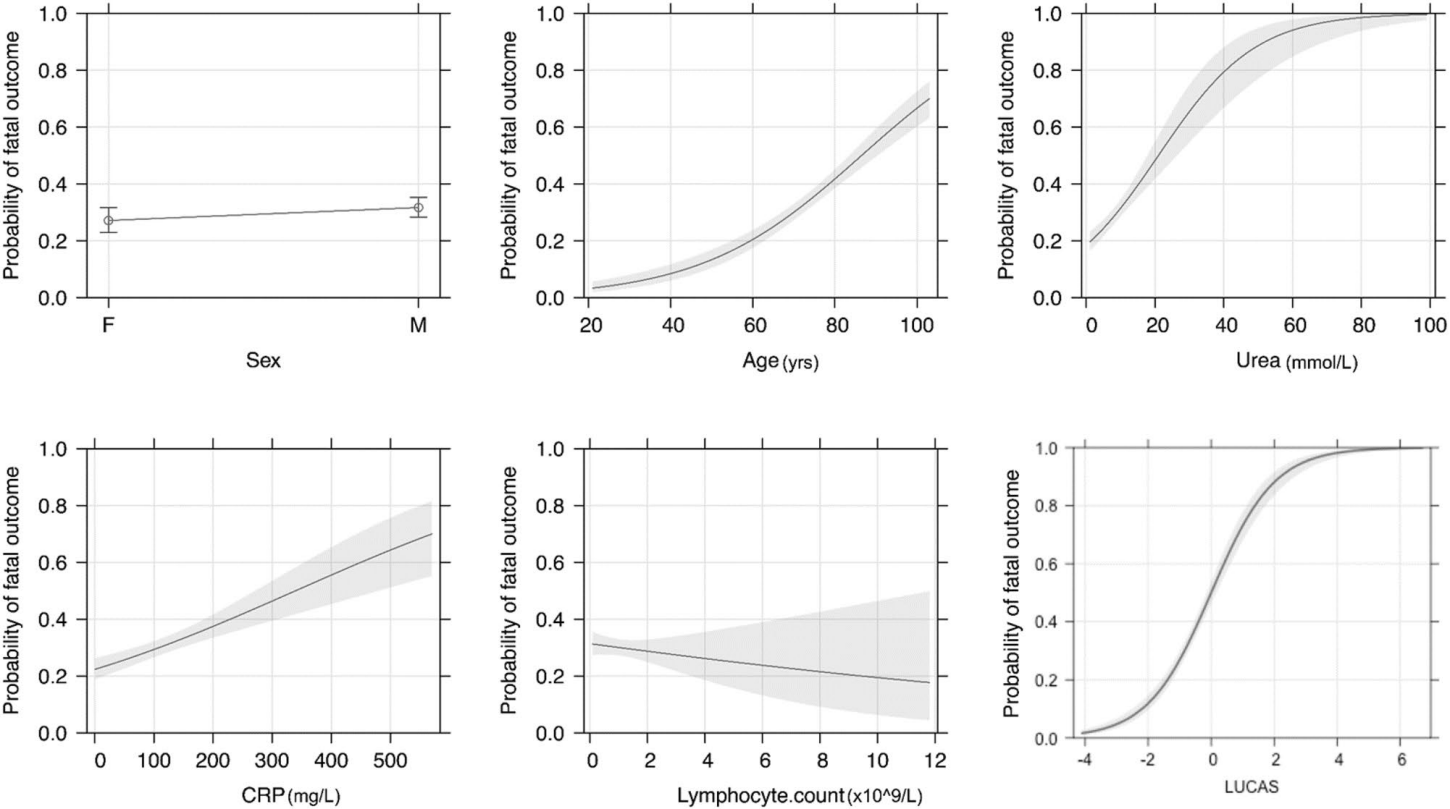
Predictor plots; multivariate association between predictors (Sex, Age, Urea, CRP, Lymphocyte count, and their combination LUCAS) and probability of fatal outcome.

Starting with the MLR model with eight predictors, namely lymphocyte, urea, CRP, age, sex, WCC, creatinine, platelets, and using GLMNET for variable selection, a final model with 5 predictors was generated achieving AUC-ROC of 0.765 (Fig. 5). The predictors, namely Lymphocyte count, Urea, CRP, Age and Sex (LUCAS), were used in the final model with optimal accuracy results comparable to the MLR and Random Forest models with different numbers and sets of predictors included (Table 2). Sex had the greatest weight (0.22) in the model. The predicted probability of 60-day in-hospital mortality can be measured by the following expression:

$$P_{Mortality} = 1 - \frac{1}{{1 + e^{{ - 5.54 + {\text{Age}}*0.05 + {\text{Sex}}*0.22 + {\text{Urea}}*0.07 + {\text{CRP}}*0.0037 - {\text{Lymphocyte}}*0.06}} }}.$$

In the Sex category, values of 0 and 1 were used for Female and Male, respectively.

From our development cohort, the LUCAS model can accurately predict 93% of the true fatal outcomes and 33% of the true non-fatal outcomes. The confusion matrices for all three prediction models are presented in Supplementary Fig. 2 (with bootstrapping over 10 repetitions). Each entry in the confusion matrix represents the percentual average cell counts across resamples. Calibration measures are similar in all three models, with LUCAS model demonstrating the best calibration performance (Supplementary Fig. 3 and Table 2).

The first step taken to stratify the outcomes from each predictor involved effect plots along with the estimated linear predictor (linear combination of the predictors with the estimated coefficients of MLR) (Fig. 5). Each sub-plot in Fig. 5 indicates how the individual predictor is related to the probability of fatal outcome. The binary predictor of sex shows an increase in probability of fatal outcome for males. For the continuous predictors (age, urea, and CRP), we can see a clear increase in the probability of fatal outcome corresponding to the increase in value of these predictors. We can also observe that in the presence of other predictors, lymphocyte count is a weak predictor; there is a slight decrease in the probability of fatal outcome with the increase in lymphocyte count. We can also notice that the error band around the mean effects is quite wide on the higher values of CRP and lymphocyte count, which is most likely due to the small number of observations in the higher ranges of these two predictors. However, when taken together in the linear combination given by LUCAS, the MLR prediction curve has a steep slope with a very narrow error bar, which indicates a very robust and accurate mortality score.

Along with the exact values of the probability of fatal outcome based on the five predictors, we propose a risk stratification (Table 3) based on these probabilities, which allows a simplified interpretation of mortality to low, medium, high, and very high risk of a fatal outcome.

**Table 3:** Risk stratification of LUCAS.

Risk level	Probability of fatal outcome cut-off	Interpretation (60-day mortality)	Proportion in development set *n* (%)	Proportion in internal validation set *n* (%)	Proportion in external validation set *n* (%)
Low	< 0.235	0.8–1.2%	574 (40)	127 (41)	201 (27)
Moderate	0.235–0.465	33–40%	516 (36)	112 (36)	333 (45)
High	0.465–0.7	53–60%	258 (18)	59 (19)	170 (23)
Very high	> 0.7	> 65%	86 (6)	12 (4)	37 (5)

Though the percentage of missing predictors in our reduced models were negligible, sensitivity analysis was performed using complete case data and as expected the model performance did not change. We also note that the simple logistic regression was preferred over machine learning approaches such as Random Forest starting from same set of predictors, based on simplicity and their performance in the validation dataset.

### Model validation

We have performed validation on an internal validation (NCCID Data between 1st May 2020 and 7th December 2020) cohort and external validation cohort consisting of data from a separate A&E site (n = 1012; between 1st March 2020 and 21st August 2020) with a small overlap with the available predictors from the derivation and internal validation cohort. However, all five predictors from the proposed model were present (LUCAS: Lymphocyte count, Urea, CRP, Age and Sex).

The median age of patients from the internal validation dataset was 74 years (IQR 62–84), which was not significantly different from the external validation dataset with a median of 76 years (IQR 61–84). The proportion of male patients was similar in both groups; 58% male in the internal validation set, and 55% male in external validation set (Table 1).

The performance of the LUCAS calculator on the internal validation cohort was very good; AUC-ROC 0.744 (CI: 0.673–0.808) with an accuracy 0.796 at the standard cut-off of 0.50 for the probability of a SARS-CoV-2 positive patient dying within 60 days of a positive test (Table 4). Use of the available NEWS2 data in combination with LUCAS had little effect on accuracy (AUC-ROC 0.747, CI: 0.668–0.821). In contrast, inclusion of CXR data in combination with LUCAS led to an increase in AUC-ROC to 0.770 (CI: 0.695–0.836). The choice to use the CXR outcome (normal vs abnormal) was therefore included in the simple LUCAS calculator as an optional extra predictor, as follows:

$$P_{Mortality} = 1 - \frac{1}{{1 + e^{{ - 6.19 + {\text{Age}}*0.05 + {\text{Sex}}*0.13 + {\text{Urea}}*0.07 + {\text{CRP}}*0.0038 - {\text{Lymphocyte}}*0.07 + {\text{CXR}}*0.55}} }}.$$

**Table 4:** Internal and external validation results.

Model	AUC-ROC	95% confidence interval	Accuracy at 0.50 cut-off	Brier score
**Development (data between 23/01/2020 and 30/04/2020) *n* = 1434**				
LUCAS	0.765	0.738–0.790	0.726	0.179
CXR	0.550	0.527–0.572	0.451	0.219
NEWS2	0.591	0.555–0.629	0.691	0.211
LUCAS + CXR	0.774	0.748–0.802	0.732	0.175
LUCAS + NEWS2	0.771	0.742–0.799	0.751	0.173
LUCAS + NEWS2 + CXR	0.779	0.746–0.808	0.742	0.171
**Internal validation (data between 01/05/2020 and 07/12/2020) *n* = 310**				
LUCAS	0.744	0.673–0.808	0.796	0.156
CXR	0.596	0.528–0.653	0.447	0.165
NEWS2	0.655	0.570–0.736	0.711	0.159
LUCAS + CXR	0.770	0.695–0.836	0.794	0.152
LUCAS + NEWS2	0.747	0.668–0.821	0.778	0.154
LUCAS + NEWS2 + CXR	0.757	0.670–0.831	0.759	0.158
**External validation (data between 01/03/2020 and 21/08/2020) *n* = 741**				
LUCAS	0.752	0.713–0.790	0.706	0.187
CXR	0.517	0.476–0.559	0.448	0.179
LUCAS + CXR	0.791	0.746–0.833	0.714	0.165

LUCAS, model using Lymphocytes, Urea, CRP, Age, and Sex; CXR, chest X-ray severity score; NEWS2, National Early Warning Score 2.

LUCAS showed an improved performance in the external validation cohort with an AUC-ROC value of 0.752 (CI: 0.713–0.790) compared to the internal dataset (AUC-ROC 0.744, CI: 0.673–0.808). By including CXR information, there is an improved prediction performance, with an AUC-ROC of 0.791 (CI: 0.746–0.833). Note that the NEWS2 score was not available for the external validation cohort, so this result could not be externally validated.

## Discussion

We have developed and validated a simplified, fast-track mortality calculator based on three rapid and routine blood parameter measurements, age and sex, with the option to use CXR results. The LUCAS calculator is freely available, relies on objective measurements only, and has been both internally and externally validated. The primary intended use of the LUCAS calculator is to aid triage on patient admission to A&E following a positive SARS-CoV-2 RT-PCR test. The robustness and generalised results in the validation process classify the tool as an excellent candidate for risk management of the mortality level in a 60-day survival interval of adult SARS-CoV-2 positive patients. The LUCAS calculator delivered higher accuracy of the external validation compared with the internal validation set, which indicates a high level of generalisation. In addition, the incorporation of CXR results as normal versus abnormal improved the prediction performance.

The NCCID dataset collected 38 clinical data points for each patient which served as candidate predictors. These predictors were categorised into demographics, risk factors, past medical history (PMH), medications, clinical observations, chest-imaging data, and laboratory parameters measured on admission to hospital. All laboratory tests were measured before the SARS-CoV-2 RT-PCR test result and mortality date, which ensured these potential predictors were blinded to the outcome. The SARS-CoV-2 swab and RT-PCR results established the final COVID-19 status. Moreover, thorough study of other included measures, such as the NEWS2 score^11^, was also performed. As a result, a comprehensive study and evaluation of all the possible blood markers and clinical patient information were taken into consideration. This study shows that a simple and objective tool can risk stratify SARS-CoV-2 positive patients within one hour after hospital admission. The primary objective showed that rapid and routine laboratory blood tests and chest imaging data added predictive value beyond the RT-PCR test and clinical observations with high AUC-ROC.

The ability of the LUCAS calculator to predict future outcomes was evaluated by non-randomly splitting the NCCID dataset to train on admissions before 30th April 2020 and predicting the outcomes for patients admitted on or after 1st May 2020. The high prediction results of LUCAS in the internal validation dataset, as well as in the external validation dataset from a different NHS site (between 1st March 2020 and 21st August 2020), demonstrates the model’s robust and generalised performance.

### Comparison with other studies

There have been many prognostic tools published, most notably the 4C Mortality Score^32^ and QCOVID^33^, which included large number of predictors in their algorithms. Our study is the first to combine a minimum number of blood results along with CXR data, to generate a simplified calculator based on as few objective predictors as possible.

The 4C Mortality Score includes 8 parameters including PMH, demographics and blood measurables, resulting in a higher AUC-ROC of 0.790^32^. However, gaining an accurate past medical history during triage is not always practical, and the 4C calculator was not externally validated. Our aim was to use the minimum number of predictors without losing accuracy, which was achieved using LUCAS that exhibits a similar level of prediction as the more complex and detailed 4C algorithm. The primary QCOVID score was developed as a risk prediction algorithm to estimate hospital admission and mortality outcomes, which also included large number of predictors including PMH^33^.

Numerous prediction models have been developed to aid triage and research into COVID-19 disease severity. While a great deal of useful insight into the disease has been gained from these studies and prognostic tools, there is a range of outcomes mostly due to some having a high risk of bias, lack of transparency or lack of internal^34^ or external validation^32,35^. Our study improves on these issues by conforming to the Prediction model Risk of Bias Assessment Tool (PROBAST)^21^ and being both internally validated from the same large NCCID dataset and externally validated in a smaller, separate hospital database. In addition, many studies require past medical history, or base the prediction on the underlying health conditions of the patient^35,36^. These data may be difficult to assess accurately on admission to hospital and may mislead should the patient have undiagnosed conditions. For this reason, we focused our model on measurables taken routinely on admission.

The NEWS2 score has been used routinely in hospitals to detect clinical deterioration, although it has mixed results in its success. In one multicentre retrospective study involving the inclusion of data from 1263 patients, the NEWS2 score was used to predict mortality, ICU admission and hospital mortality and resulted in an AUC-ROC of 0.65 for 30-day mortality^9^. This compares to our NEWS2 findings of an AUC-ROC 0.59 in development cohort and 0.66 in internal validation cohort. When we combined NEWS2 with LUCAS in predicting mortality within 60 days, this increased the AUC-ROC to 0.77 in development cohort and 0.75 in internal validation cohort, thus indicating an improvement of accuracy when in combination with the LUCAS algorithm. This large increase in predictive power also gives weight to the use of LUCAS over NEWS2 in prognosis modelling for COVID-19.

All the predictors used in the LUCAS calculator have been shown to be useful predictors in other published studies. Lymphocyte count^17,20,37^, urea^32,38^, and CRP^39–44^ are recognised as key measurable predictors of severity of SARS-CoV-2 infection, and age and sex are also well-known predictors of mortality^32,33,38,43^. While these factors have been used in other prediction models, our study is the first to use only these predictors in a prognostic score along with the option to use CXR data.

Inclusion of CXR data is optional for the online LUCAS calculator and based on simple outcome of normal/abnormal image results. The ability to include CXR results is not widely available in other prediction calculators and has been included in a study^35^ along with ten other parameters (symptoms, past medical history and measurables). More recently, some of the studies have included the CXR imaging in prognostic models^45,46^, with good accuracy; however, they have either utilised information such as electronic health records^45^ including comorbidities^46,47^, which are not always known at the point of care, additional blood biomarkers such as D-Dimer^7,41^ and lactate dehydrogenase^42^, which are not measured routinely during triage, or incorporated complex deep-learning methodologies^46^, affecting the explainability and simplicity of the model. Indeed, in a parallel study, we have developed a highly accurate deep-learning based model (DenResCov-19) to classify from CXR images patients positive for SARS-CoV-2, tuberculosis, and other forms of pneumonia^6^, which will be integrated into the LUCAS calculator in a future study. Our focus in this study, however, was to form a simplified model on rapid and routine blood test results, with the option to use CXR images, which we have achieved.

Throughout the study, we have carefully considered the risk of bias that is inherent in retrospective studies. By conducting both internal and external validation, the study here indicates a robust model with reduced bias, since only patients testing positive for SARS-CoV-2 were included in the development of the LUCAS algorithm. The size of the external validation set was smaller than the development set allowing us to check for discrimination of population size, and the results indicate that the LUCAS calculator can predict from small cohorts as well as it can from larger size populations.

The patient data was collected at an early stage of the pandemic when treatments differed compared to later in the year, which would affect the death rate in hospital. In addition, our results do not account for non-hospital deaths or deaths outside the 60-day window following diagnosis. Over time, any algorithm of mortality will change due to improvements in therapies as well as the use of vaccination which will change the profile of those at risk of COVID-19 related death^48^. While these changes in therapeutic interventions change over time^49^, multiple studies have reported the associated changes to inflammatory markers that are found in severe cases of COVID-19^39^. While improvements in medical care have significantly reduced mortality, the immunological responses that indicate severe cases of COVID-19 have not changed, making the use of prediction modelling important to aid in the triage of patients.

## Conclusion

The major strengths of this study in mortality prediction of COVID-19 patients include the analytical approach taken, the comparison with the widely used NEWS2 score, the inclusion of CXR data, and the evaluation in both internal and external validation cohorts. The CXR data contributes to an improvement in the mortality prediction of the patient, although the CXR information used in the LUCAS calculator is binary (normal or abnormal). Further development of this work is currently ongoing, which will extract CXR image features using machine learning or deep learning methods^6^ and combine with LUCAS to deliver an automated, integrated biomarker-imaging analysis.

## Supplementary Information

Supplementary Information.

## Data Availability

The data that support the findings of this study are available from the NCCID^23^ and another NHS site; but restrictions apply to the availability of these datasets, which were used under license for the current study, and are not publicly available.

## Code availability

The Simple Logistic Regression based calculator is freely available at https://mdscore.org/.
