# Supplementary material for: A robust COVID-19 mortality prediction calculator based on Lymphocyte count, Urea, C-Reactive Protein, Age and Sex (LUCAS) with chest X-rays
Source: Sci Rep. 2022 Oct 29;12:18220. doi: 10.1038/s41598-022-21803-2 (PMC9617052; doi:10.1038/s41598-022-21803-2)
Supplement: Supplementary file 1 — Supplementary Information. [file 41598_2022_21803_MOESM1_ESM.pdf]

Supplementary Table 1. Origins of Dataset: development and Internal Validation (NCCID NHS Trust Summary) https://nhsx.github.io/covid-chest-imaging-database/

	Total	Male	Female	Dead	Alive	Prop. dead
**Ashford and St Peters Hospitals NHS Foundation Trust**	139	59	80	133	6	0.96
**Betsi Cadwaladr University Health Board**	114	67	47	41	73	0.36
**Brighton and Sussex University Hospitals NHS Trust**	248	136	112	99	149	0.40
**Cambridge University Hospitals NHS Foundation Trust**	0	0	0	0	0	NA
**Cwm Taf Morgannwg University Health Board**	90	51	39	24	66	0.27
**George Eliot Hospital NHS Trust**	665	394	271	234	431	0.35
**Hampshire Hospitals NHS Foundation Trust**	205	117	88	50	155	0.24
**Imperial College Healthcare NHS Trust**	1,708	1,042	666	456	1,252	0.27
**Liverpool Heart and Chest NHS Foundation Trust**	61	53	8	26	35	0.43
**London North West University Healthcare NHS Trust**	420	227	193	149	271	0.35
**Norfolk and Norwich University Hospitals NHS Foundation Trust**	692	384	308	238	454	0.34
**Oxford University Hospitals NHS Foundation Trust**	24	15	9	14	10	0.58
**Royal Cornwall Hospitals NHS Trust**	759	523	236	300	459	0.40
**Royal Surrey NHS Foundation Trust**	1	0	1	1	0	1.00
**Royal United Hospitals Bath NHS Foundation Trust**	1,533	987	546	417	1,116	0.27
**Sandwell and West Birmingham Hospitals NHS Trust**	0	0	0	0	0	NaN
**Sheffield Childrens NHS Foundation Trust**	1	1	0	0	1	0.00
**Taunton and Somerset NHS Foundation Trust**	56	44	12	16	40	0.29
**West Suffolk NHS Foundation Trust**	515	330	185	195	320	0.38

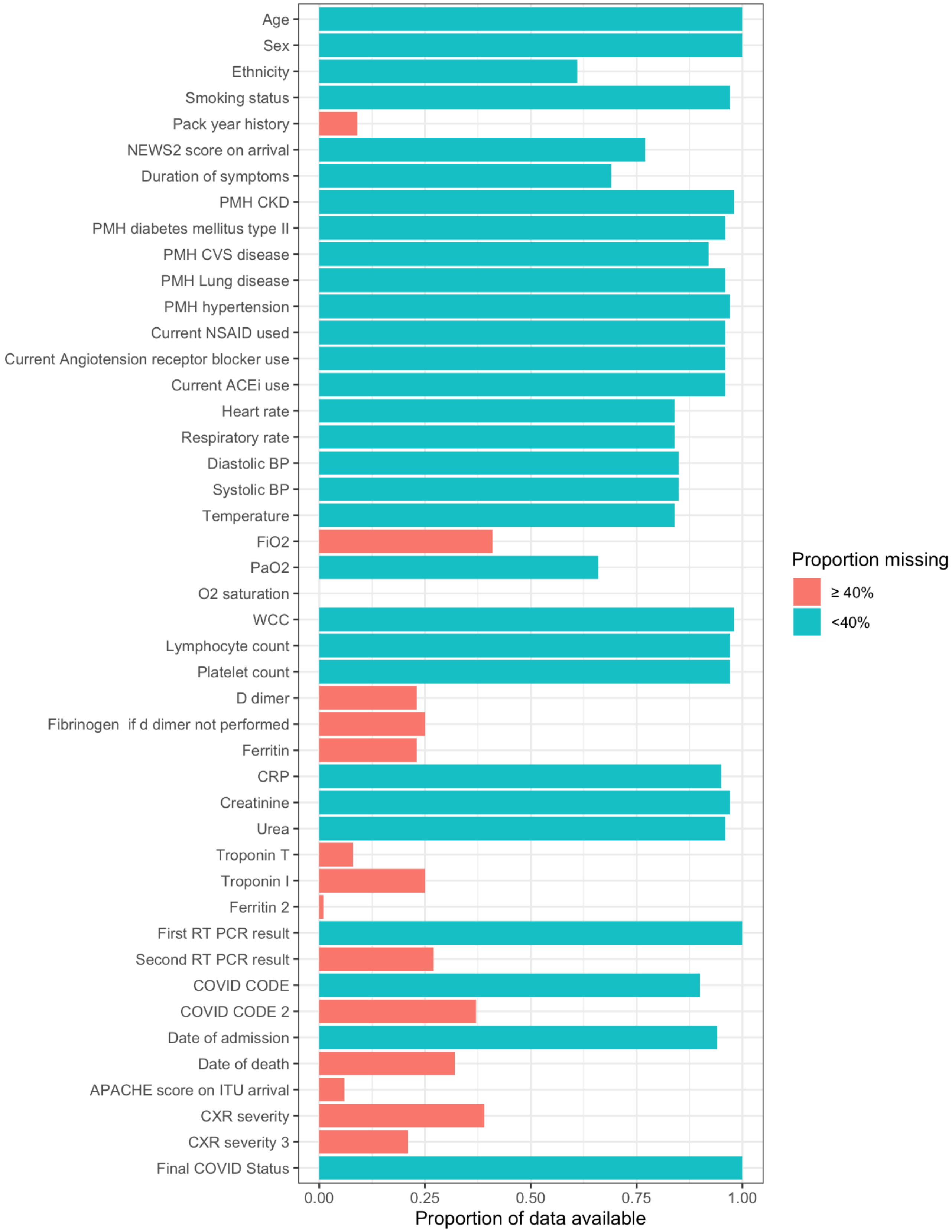

Supplementary Figure 1. Density map of data points from the NCCID cohort to determine predictors to be used in both Development and Internal Validation dataset. Predictors with available data above the 60% threshold were used in the analysis.

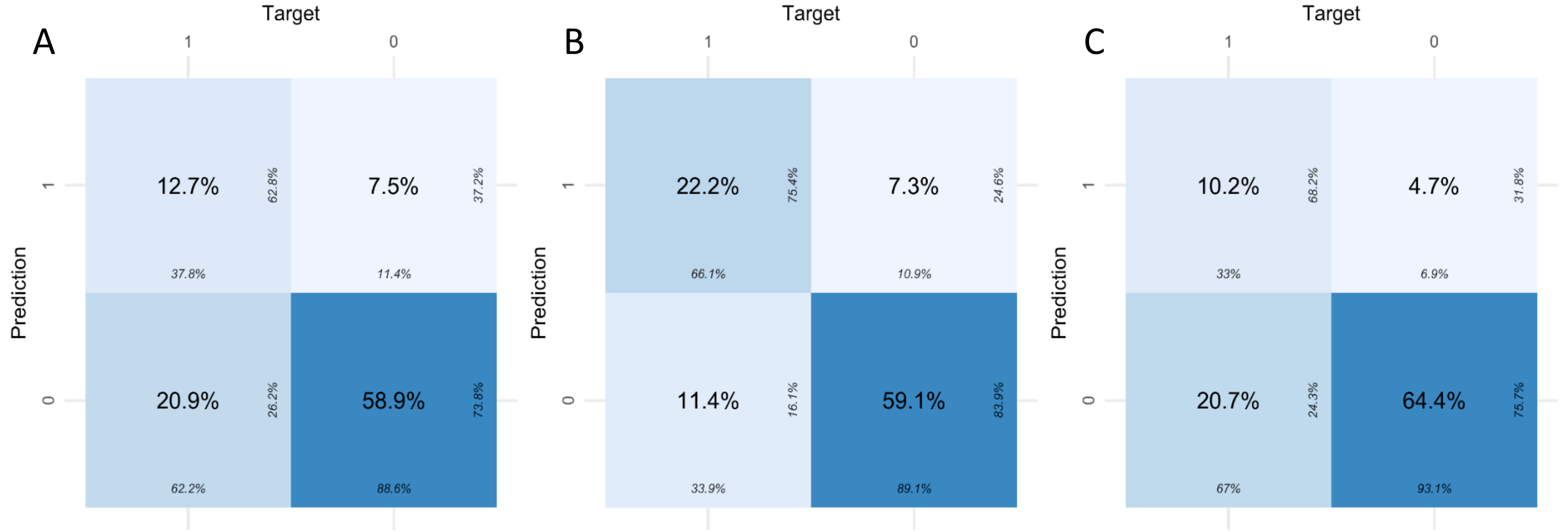

Supplementary Figure 2. Confusion Matrix for A. Multivariable Logistic regression (MLR) model, B. Random Forest model and C. LUCAS calculator model.

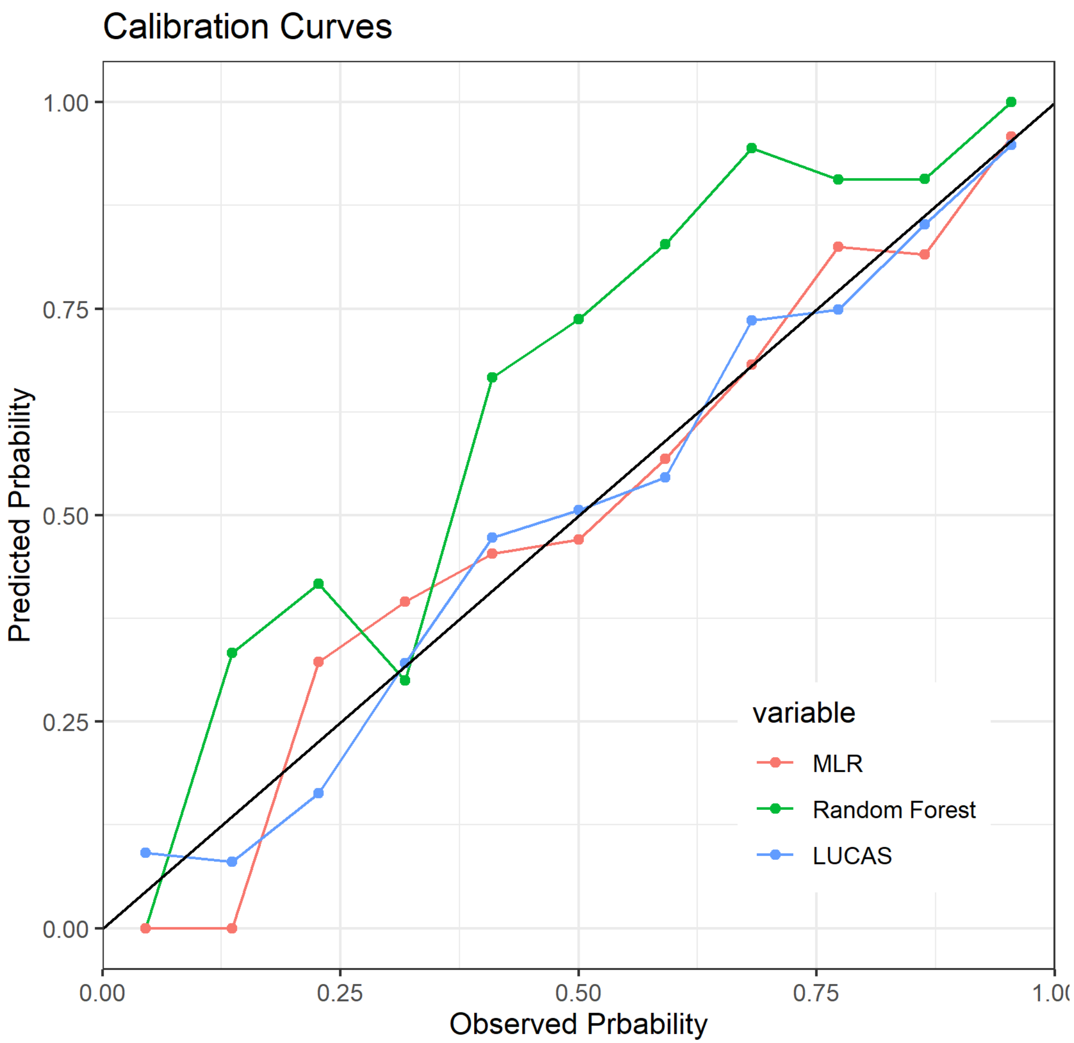

Supplementary Figure 3. Calibration curves for the Multivariable Logistic regression (MLR), Random Forest, and LUCAS model.
